# Real-World Evaluation of Next-Generation Sequencing in Lung Cancer: Associations Between Histological Subtypes and Genomic Alterations

**DOI:** 10.3390/cimb48060628

**Published:** 2026-06-16

**Authors:** Javier Azúa-Romeo, Maria Cabetas, Irene Rodriguez, Bárbara Angulo, Arantxa Andueza

**Affiliations:** 1 Pathology Department, Analiza, 28085 Madrid, Spain; 2 Department of Anatomy and Histology, University of Zaragoza, 50006 Zaragoza, Spain; 3 Facultad de Medicina, Universidad de Zaragoza, 50006 Zaragoza, Spain; 4 Molecular Diagnostic Department, Analiza, 28085 Madrid, Spain

**Keywords:** NGS, lung cancer, *KRAS*, *EGFR*, PD-L1

## Abstract

Background: Lung cancer is a highly heterogeneous disease in which molecular characterization has become essential for guiding personalized therapies. The implementation of next-generation sequencing (NGS) allows for the simultaneous detection of multiple genomic alterations, improving tumor profiling and therapeutic decision-making. This study aimed to characterize the molecular landscape of lung cancer using NGS and to evaluate its association with histological subtypes and programmed death-ligand 1 (PD-L1) expression. Methods: A retrospective observational study was conducted on 96 patients diagnosed with lung cancer between 2023 and 2025. Molecular profiling was performed using the Action OncoKitDx panel. Associations between genetic alterations, histological subtypes, and PD-L1 expression were analyzed using Fisher’s exact test, with *p* < 0.05 considered statistically significant. Results: Adenocarcinoma was the most common histological subtype (67.7%), followed by squamous cell carcinoma (26%). The most common mutations were *KRAS* (34.4%), *TP53* (29.2%), and *EGFR* (11.5%). *KRAS* mutations were significantly associated with adenocarcinoma (*p* = 0.001), while squamous cell carcinoma showed a higher frequency of cases without molecular alterations detected by the NGS panel (*p* = 0.002). Co-mutations were identified in 22.9% of cases, with *KRAS*–*TP53* being the most common combination. Tumors harboring *EGFR* mutations showed a significantly lower frequency of co-mutations (*p* = 0.012). No significant associations were found between *PD-L1* expression and either histological subtypes or the analyzed genetic alterations. Conclusions: Lung cancer exhibits marked molecular heterogeneity, with a predominance of *KRAS* mutations in adenocarcinoma. The low frequency of co-mutations in *EGFR*-mutated tumors supports their role as dominant driver alterations. The lack of association between *PD-L1* expression and genomic alterations highlights the complexity of its regulation and suggests the involvement of multiple biological factors. These findings reinforce the clinical value of NGS in comprehensive tumor profiling and in the development of precision medicine strategies.

## 1. Introduction

Lung cancer is one of the leading public health problems worldwide. According to data from the Global Cancer Observatory (GLOBOCAN), in 2022, there were an estimated 2.48 million new cases and more than 1.8 million deaths, making it the most prevalent cancer in the world and the leading cause of cancer mortality globally. It is the most common cancer in men and the second most common in women, behind breast cancer. Smoking remains the primary risk factor for lung cancer. Changes in smoking patterns over recent decades have contributed to a gradual narrowing of the incidence gap between men and women [1].

In this context, lung cancer should not be considered a single entity, but rather a heterogeneous group of neoplasms with different histological characteristics and clinical behavior. Traditionally, it has been classified into non-small-cell lung cancer (NSCLC) and small-cell lung cancer (SCLC), with the former being the most common. Within NSCLC, the most common subtypes include adenocarcinoma and squamous cell carcinoma, in addition to less frequent ones such as adenosquamous carcinoma, large cell carcinoma, or anaplastic carcinoma. On the other hand, SCLC corresponds to high-grade neuroendocrine tumors with aggressive clinical behavior. In some cases, especially with limited samples, it is not possible to precisely determine the histological subtype, and the category “Not Otherwise Specified” (NOS) is used [2,3,4].

The characterization of these subtypes is based on histopathological examination supported by immunohistochemistry (IHC) techniques, which allow for the identification of specific marker expression and improve diagnostic accuracy, particularly in small or poorly differentiated samples. In this regard, markers such as TTF-1 and Napsin A are associated with adenocarcinomatous differentiation, while p40 and p63 are characteristic of squamous cell carcinoma; generally, these profiles show differential expression that facilitates their distinction. In cases where characteristics of both lineages coexist, a diagnosis of adenosquamous carcinoma may be considered, always in correlation with morphological findings. On the other hand, neuroendocrine tumors show expression of markers such as synaptophysin, chromogranin, and CD56 [3,5].

Until a few years ago, tumor characterization in lung cancer was based primarily on histological and immunohistochemical studies, with the latter being particularly relevant for the selection of immunotherapy treatments through the evaluation of biomarkers such as PD-L1. However, in recent years, the understanding of molecular alterations involved in lung cancer has become fundamental for its clinical management. Mutations in genes such as *KRAS*, *EGFR*, or *TP53* act as oncogenic drivers and have both prognostic and therapeutic implications, enabling the identification of targets for targeted therapies. Overall, molecular characterization of the tumor is essential for the implementation of personalized medicine strategies [3,6,7].

In this context, Next-Generation Sequencing (NGS) has become a fundamental tool for the simultaneous analysis of multiple genetic alterations, enabling a more comprehensive molecular characterization of tumors in routine clinical practice. Although several molecular patterns in lung cancer have already been described, additional real-world studies are necessary to validate the reproducibility and clinical applicability of these findings across heterogeneous patient populations. Therefore, the objective of this study was not only to describe the molecular profile of lung cancer patients using NGS but also to evaluate the utility of targeted NGS panels in a real-world clinical cohort and to assess their relationship with histological subtypes and PD-L1 expression in routine diagnostic practice [3,5,6,7].

## 2. Materials and Methods

A retrospective observational study was conducted by selecting patients from the Analiza reference laboratory. Specifically, the cohort consists of patients diagnosed with various histological subtypes of lung cancer at Analiza over the past three years (2023–2025). Of all patients, only those who underwent NGS for molecular tumor characterization and who had been previously diagnosed at that laboratory were selected.

To filter all cases and select those of interest, Analiza’s Laboratory Information System (LIS) for Pathology, Atlas, was used. An initial filter was applied based on the SNOMED topographic code, selecting only cases where this field indicated “lung”; subsequently, only those classified as malignant and coded in SNOMED as “malignant pathology,” “adenocarcinoma,” “carcinoma,” “small cell carcinoma,” “squamous cell carcinoma,” and “oat cell carcinoma,” among others. In this way, the total number of malignant lung neoplasms diagnosed each year was obtained.

After filtering the cases according to the parameters mentioned above, 126 cases were obtained for the study. From this point, certain inclusion and exclusion criteria were applied; initially, cases were excluded in which the analyzed neoplasms corresponded to metastases from primary tumors in other organs rather than primary lung tumors (11 cases excluded, leaving a sample size of 114); since the inclusion of such cases would not be appropriate for this study, metastatic tumors of extrapulmonary origin present distinct molecular profiles determined by the tissue of origin, which could introduce bias into the specific molecular characterization of lung cancer and affect the validity of the results.

Thirteen cases were excluded due to insufficient tumor material, including samples in which molecular analysis could not be adequately performed or cases that yielded non-conclusive NGS results because of insufficient tumor material quality or quantity. Cases with unsuccessful or non-conclusive sequencing results due to insufficient material were included within this excluded group. Finally, cases in which PD-L1 was not analyzed were also excluded (5 cases excluded), due to insufficient biopsy size to perform both NGS and PD-L1 testing, in which NGS was prioritized. Applying these criteria resulted in a final sample size of 96 cases.

Part of the cohort included in the present study was previously described in a preliminary preprint report from our group. However, the current study applies revised inclusion and exclusion criteria, resulting in a refined cohort and incorporating additional statistical analyses evaluating associations between molecular alterations, histological subtype, co-mutation patterns, and PD-L1 expression.

All samples corresponded to routine diagnostic FFPE tissue specimens that were reviewed by pathologists prior to molecular analysis as part of routine diagnostic practice. Cases with insufficient tumor material or inadequate sample quality for NGS testing were excluded from the study.

This study utilized the Action OncoKitDx high-throughput sequencing panel (Health in Code Group, Valencia, Spain) in conjunction with the NextSeq 550 platform (Illumina, San Diego, CA 92122, USA) to identify relevant genetic alterations in solid tumors by analyzing a set of genes involved in oncogenesis. The process began with DNA extraction from formalin-fixed, paraffin-embedded (FFPE) tissue samples. QIAamp DSP DNA FFPE Tissue kit (QIAGEN GmbH, Tuebingen, Germany) was used for DNA extraction according to the manufacturer’s recommendations. After quantification by Qubit 4 Fluorometer (Invitrogen, Thermo Fisher, Waltham, MA, USA), an input of 200–300 ng of DNA was ensured. DNA quality was assessed by DIN value (DNA Integrity Number) using Tape Station with Genomic DNA Reagents and Screen Tape (Agilent, Santa Clara, CA, USA). The recommended DIN value is >3. While the NGS protocol is feasible in samples with DIN < 3, sensitivity for genetic alteration detection may be reduced. Subsequently, the genetic material underwent enzymatic fragmentation and a process of target region enrichment via capture with specific probes. Finally, sequencing was performed using reversible terminator synthesis technology on the aforementioned platform, enabling the precise identification of genomic alterations of interest.

The panel used enables the detection of various types of genetic alterations, including point variants (substitutions, insertions, and deletions), copy number variations (CNVs), and structural rearrangements. These alterations have both diagnostic and prognostic relevance, as well as therapeutic implications, as they constitute potential targets for targeted therapies or predictive biomarkers of response. Furthermore, the panel includes analysis of microsatellite instability (MSI), which is useful for selecting immunotherapies, as well as the study of pharmacogenetic variants related to the efficacy and toxicity of certain chemotherapeutic agents. Taken together, this approach enables a comprehensive molecular characterization of the tumor, facilitating therapeutic decision-making and optimizing the clinical management of cancer patients.

The Action OncoKitDx panel includes [2]:

Whole-exome sequencing of 55 genes: *ALK*, *ARID1A*, *ATM*, *ATRX*, *BAP1*, *BRAF*, *BRCA1*, *BRCA2*, *CHEK2*, *CDH1*, *CTNNB1*, *EGFR*, *ERBB2*, *ESR1*, *FGFR1*, *FGFR2*, *FGFR3*, *FGFR4*, *GNA11*, *GNAQ*, *H3F3A*, *HIST1H3B*, *HIST1H3H*, *HRAS*, *IDH1*, *IDH2*, *KIT*, *KRAS*, *MAP2K1*, *MET*, *MLH1*, *MSH2*, *MSH6*, *MTOR*, *MYC*, *NRAS*, *NTRK1*, *NTRK2*, *NTRK3*, *PALB2*, *PBRM1*, *PDGFRA*, *PIK3CA*, *PMS2 + 5′UTR*, *PTEN*, *POLD1*, *POLE*, *RET*, *ROS1*, *SDHA*, *SDHB*, *SDHD*, *TERT + 5′UTR* and *TP53*.

Sequencing of hotspot regions in the *TSC1*, *TSC2*, and *AKT1* genes.

Analysis of rearrangements in the *ALK*, *BRAF*, *EGFR*, *FGFR2*, *FGFR3*, *NRG1*, *NTRK1*, *NTRK2*, *NTRK3*, *RET*, and *ROS1* genes. The Action OncoKitDx panel uses probes that cover the intronic regions where breakpoints have been most frequently identified: intron 19 of *ALK*; introns 31–35 of *ROS1*; introns 9–11 of *RET*; intron 17 and 3′UTR of *FGFR2*; *FGFR3* intron 17 and 3′UTR; *NTRK1* introns 8–12; *NTRK2* introns 10 and 12; *BRAF* introns 7–10; and *EGFR* introns 7, 23, 24, and 25.

Microsatellite instability (MSI) analysis using a panel of 110 microsatellite regions.

Detection of CNVs (amplifications and deletions) in genes covered by the panel and analysis of large chromosomal alterations across the entire genome, including deletions or gains of entire chromosomes or chromosomal regions.

Detection of variants related to the patient’s pharmacogenetics to assess response or toxicity to chemotherapy treatments. Variants are analyzed in seven genes that affect response to treatments for tumors of different origins: DPYD (rs3918290, rs67376798, rs55886062, rs115232898, rs75017182), XRCC1 (rs25487), UGT1A1 (rs4148323), CYP2D6 (rs3892097, rs5030655), MTHFR (rs1801133), TPMT (rs1142345, rs1800460, rs1800584, rs1800462), and CYP2C9 (rs1799853, rs1057910).

Bioinformatic processing of the obtained sequences was performed using the Data Genomics platform, which allows for alignment with the reference genome and subsequent identification of variants through the application of quality filters. Both the panel used and the analysis software are certified for in vitro diagnostic use. The system enables the detection of point mutations with a minimum allelic frequency of 5%, provided that the samples have at least 30% tumor cell content and a sequencing depth greater than 200 reads. It should be noted that the use of next-generation sequencing for comprehensive molecular profiling of tumors is in accordance with the recommendations of the European Society for Medical Oncology (ESMO) [2,8,9,10].

PD-L1 expression was categorized according to tumor proportion score (TPS) as negative (<1%), low expression (1–49%), and high expression (≥50%), following commonly used clinical thresholds in non-small-cell lung cancer.

Statistical analysis was performed using the Jamovi software (version 2.7.15.0). Categorical variables were analyzed using Fisher’s exact test, due to the small sample size (96 cases) and the fact that the frequency of some categories was less than 5 cases for some variables. A *p*-value < 0.05 was considered statistically significant for assessing the association between the studied variables. Additionally, a multivariate binomial logistic regression analysis was performed to evaluate whether *KRAS*, *TP53*, *EGFR* alterations and histological subtype were independently associated with PD-L1 expression. PD-L1 expression was dichotomized as negative (<1%) versus positive (≥1%).

Given the exploratory nature of the study and the limited sample size, no formal correction for multiple comparisons was applied. Therefore, the reported *p*-values should be interpreted as exploratory and hypothesis-generating.

## 3. Results

The final sample size consisted of 96 cases, which were classified according to histological subtype (adenocarcinoma, squamous cell carcinoma, adenosquamous carcinoma, neuroendocrine carcinoma, anaplastic carcinoma, and “Not Otherwise Specified” (NOS)). The distribution of cases is shown in Table 1.

The distribution of molecular alterations detected by NGS was evaluated in relation to histological subtype. The results are shown in Table 2.

This initial analysis revealed that the most common histological subtype is adenocarcinoma (67.71% of cases), followed by squamous cell carcinoma (26.04% of cases); in contrast, the remaining subtypes of NSCLC, as well as small-cell lung cancer (SCLC) and neuroendocrine carcinoma, are much less common, with only isolated cases found.

Regarding the mutations analyzed by NGS, the most frequent were *KRAS* (34.38% of cases), *TP53* (29.17% of cases), and *EGFR* (11.46% of cases). The remaining alterations were rare (*BRAF*, *FGFR1*, *ALK*, *MDM2*, *MET*, *PTEN*, *ARID1A*, *PIK3CA*, *TSC1*, *ATM*, *AKT*).

After analyzing an overview of the data obtained, a more comprehensive statistical analysis was performed based on the altered genes and histological subtypes, which are presented below.

The *KRAS* mutation showed a significant association with histological type (*p* = 0.001), being more frequent in adenocarcinoma (Table 3). The absence of mutations also showed a significant association (*p* = 0.002), with a higher proportion in squamous cell carcinomas (Table 4). The remaining mutations did not show significant associations with any of the histological subtypes analyzed (Appendix A, Table A1, Table A2, Table A3, Table A4, Table A5, Table A6, Table A7, Table A8, Table A9, Table A10, Table A11, Table A12 and Table A13).

Regarding the number of molecular alterations per tumor, 30 cases showed no molecular alterations detected by the NGS panel, 44 cases harbored a single molecular alteration, while 22 cases (22.9%) had two or more mutations. No statistically significant differences were observed according to histology (*p* = 0.054) (Table 5).

Likewise, the association of mutations (22 cases in total) was analyzed according to the different histological subtypes listed below, of which 17 cases present co-mutations of 2 mutations (Table 6), 4 cases of co-mutations of 3 mutations (Table 7), and one case of up to 4 simultaneous mutations (Table 8).

Of all the cases studied, the most frequent co-mutation was *KRAS* + *TP53* (11 cases), predominantly in adenocarcinoma, although this was not statistically significant (*p* = 0.184) (Table 9). Furthermore, *EGFR*-mutated tumors showed a significantly lower frequency of co-mutations compared with *EGFR* wild-type tumors (Fisher’s exact test, *p* = 0.012), supporting the relative molecular exclusivity of *EGFR* alterations in this cohort (Table 10).

Following a thorough analysis of the results obtained via NGS, we also analyzed PD-L1 expression according to histological subtypes (Table 11 and Table 12). Of the total cases, 41 cases (42.7%) had negative PD-L1 expression (<1%), 42 cases (43.8%) had low PD-L1 expression (1–49%), and 13 cases (13.5%) had high PD-L1 expression (≥50%). Overall, PD-L1 expression showed a relatively balanced distribution between negative (<1%) and low/intermediate (1–49%) expression categories.

No significant associations were observed between PD-L1 expression and histological type (*p* = 0.185), nor with *TP53* (*p* = 0.744), *KRAS* (*p* = 0.508), or *EGFR* (*p* = 0.242) mutations. However, a trend toward lower PD-L1 expression was observed in tumors with *EGFR* mutations (Table 13, Table 14 and Table 15).

A multivariate binomial logistic regression analysis was subsequently performed to evaluate whether *KRAS*, *TP53*, *EGFR* alterations and histological subtype were independently associated with PD-L1 expression (Table 16). No statistically significant independent associations were observed.

## 4. Discussion

The molecular characterization of lung cancer using next-generation sequencing techniques has transformed the diagnostic and therapeutic approach to this disease. In this context, the present study provides a comprehensive view of the molecular profile in a real-world clinical cohort, highlighting distinct patterns of genetic alterations based on histological subtype and their relationship with immunological biomarkers.

One of the most relevant findings of this study is the confirmation of the strong association between *KRAS* mutations and adenocarcinoma in a real-world clinical cohort (*p* = 0.001), consistent with previously published evidence identifying this gene as one of the main molecular drivers in this histological subtype, with frequencies around 30%, and significantly more frequent in this histological subtype compared to others [10,11,12,13].

Another notable aspect is the higher proportion of tumors without detectable mutations in squamous cell carcinoma. This finding is consistent with recent studies describing that squamous cell lung carcinoma presents a distinct molecular profile, characterized by a lower frequency of classic driver mutations such as *KRAS* or *EGFR*, and greater genomic heterogeneity. Far from being interpreted as a true absence of genetic alterations, this result likely reflects the limitations of targeted sequencing panels in capturing the genomic complexity of this subtype. In this regard, squamous cell carcinoma could be characterized by a distinct molecular profile, with alterations less represented in standard panels or with alternative oncogenic mechanisms, underscoring the need for broader analytical strategies for its adequate characterization [13,14].

The remaining mutations did not show statistically significant differences across the histological subtypes analyzed; this may be due to the very small number of cases for the other subtypes because of their low prevalence (neuroendocrine c., anaplastic c.); a larger sample size would be needed to reassess this in the future. Various authors refer to the relationship between various genes such as *ALK*, *BRAF*, *ROS1*, or *MET* (associated with co-mutation alongside *MDM2*) in CPCNP tumors; however, most studies do not investigate this relationship more specifically with regard to the different histological subtypes. Some authors note that *PIK3CA*, *FGFR1*, or *SOX2* are more frequently present in squamous cell carcinoma than in adenocarcinoma, or that mutations in *JAK3*, *NRAS*, or *VHL1* are found exclusively in small cell carcinoma and not in large cell carcinoma [3,15,16,17].

Tumors with *EGFR* mutations showed a statistically significantly lower frequency of co-mutations, supporting their role as dominant molecular drivers. This finding is consistent with the literature, which describes that *EGFR* mutations act as dominant molecular drivers and often exhibit mutual exclusivity with other genetic alterations. This phenomenon of “mutual exclusivity” has been widely described in the literature, reinforcing the validity of our results and providing additional evidence in a clinical practice context. Furthermore, it is described that *EGFR* mutations exhibit a pattern of mutual exclusivity with other molecular drivers, particularly with *KRAS*, as both alterations are generally mutually exclusive. The identification of this pattern has significant implications, as it suggests that *EGFR*-mutated tumors may exhibit a more defined oncogenic dependency, which can influence both response to targeted therapies and clinical course. However, rare cases of co-mutations have been described, such as the coexistence of *EGFR* with *ALK* or *ROS1*, with very low frequencies (less than 1%), indicating that, although uncommon, the presence of concomitant alterations is possible, requiring individualized dual-targeted therapies. This fact could explain the few cases of co-mutation observed in our cohort [18,19].

The presence of multiple mutations in a significant percentage of tumors in our cohort highlights the high molecular heterogeneity of lung cancer. Although no significant differences were observed between histological subtypes in terms of the number of mutations, the identification of recurrent patterns, such as the *KRAS*–*TP53* co-mutation, suggests that genomic alterations do not occur in isolation but rather in coordinated patterns reflecting tumor biological complexity and clonal evolution. This phenomenon, widely described in high-throughput sequencing studies, may have relevant biological and clinical implications, particularly regarding response to therapies, including immunotherapy [20].

Our results highlight the tendency of *KRAS* to coexist with other alterations, particularly *TP53*, suggesting the presence of cooperative oncogenic programs, predominantly observed in adenocarcinoma, although without reaching statistical significance. This finding is consistent with the literature, which describes that the coexistence of mutations in *KRAS* and *TP53* is relatively common in lung cancer; recent studies indicate that genomic alterations in lung cancer do not occur in isolation but tend to present in patterns of co-occurrence, particularly among genes such as *EGFR*, *KRAS*, and *TP53*. Furthermore, several studies have demonstrated that these co-mutations may have relevant clinical implications, particularly regarding response to immunotherapy, suggesting a potential role as prognostic and predictive biomarkers. Although previous studies have reported associations between *KRAS*–*TP53* co-mutations, increased PD-L1 expression, and enhanced response to immunotherapy, our cohort did not demonstrate significant associations between PD-L1 expression and either *KRAS* or *TP53* alterations. This discrepancy may reflect the limited sample size of our cohort, the exploratory nature of the analyses, and the complex biological regulation of PD-L1 expression, which is likely influenced by multiple interacting molecular and microenvironmental factors [20,21,22].

No significant associations were observed between PD-L1 expression and either the histological subtypes or the genetic alterations analyzed. This finding, far from undermining the results, highlights the complexity of the regulation of this biomarker. However, the literature describes that certain genetic alterations, particularly *TP53* and *KRAS*, may be associated with higher PD-L1 expression and a higher tumor mutational burden, suggesting a more immunogenic tumor microenvironment. However, these findings are not consistent across different studies, suggesting that PD-L1 expression is influenced by multiple biological factors and not exclusively by specific genetic alterations. Differences in cohort composition, patient characteristics, and study methodologies may also contribute to the variability observed across published studies. In our cohort, these observations were further supported by multivariate analysis, in which no independent associations were identified between PD-L1 expression and *KRAS*, *TP53*, *EGFR* alterations, or histological subtype. These findings reinforce the complexity of PD-L1 regulation and suggest that its expression may depend on multiple interacting biological mechanisms beyond isolated genomic alterations [22,23].

The *KRAS*–*TP53* co-mutation was the most frequent co-mutational pattern identified in our cohort. Previous studies have suggested that tumors harboring concurrent *KRAS* and *TP53* alterations may exhibit a more inflamed tumor microenvironment, increased PD-L1 expression, and enhanced responsiveness to immune checkpoint inhibitors compared with other molecular subgroups. Although no significant association between *KRAS*, *TP53*, and PD-L1 expression was observed in our cohort, likely due to the limited sample size and lack of clinical outcome data, the high prevalence of this co-mutational pattern highlights its potential clinical relevance. Future studies integrating treatment response and survival data would be valuable to further explore the prognostic and therapeutic implications of *KRAS*–*TP53* co-mutations in routine clinical practice.

This study has several limitations that should be acknowledged. First, the retrospective single-center design and the relatively limited sample size, particularly for rare histological subtypes, may reduce the statistical power of subgroup analyses and limit the detection of statistically significant associations. The low number of cases in certain histological subtypes, particularly neuroendocrine carcinoma (N^er^ = 2), adenosquamous carcinoma (N^er^ = 2), and anaplastic carcinoma (N^er^ = 1), limits the statistical power of subgroup analyses. Therefore, findings involving these rare subtypes should be considered descriptive and interpreted with caution. In addition, the present study was based on the retrospective analysis of fully anonymized molecular diagnostic records generated during routine clinical practice. Consequently, detailed clinical and demographic variables such as age, sex, smoking history, ethnicity, tumor stage, treatment history, and other patient-related characteristics were not available for inclusion in the analysis. In particular, the absence of smoking history data represents an important limitation, as tobacco exposure is strongly associated with the molecular landscape of lung cancer, especially with alterations involving genes such as *KRAS* and *TP53*. Therefore, the potential influence of smoking-related mutational patterns could not be assessed in the present study. Furthermore, the study was based on tumor-only sequencing, and matched normal samples were not available. Therefore, germline status could not be systematically assessed, and the reported alterations correspond to variants identified in tumor samples during routine molecular profiling. In addition, DNA extracted from formalin-fixed paraffin-embedded (FFPE) tissue samples may be affected by degradation, fragmentation, and fixation-related artefacts, which could potentially influence sequencing quality and variant detection despite the quality-control measures applied during routine diagnostic testing. Additionally, although the Action OncoKitDx panel covers a broad spectrum of clinically relevant cancer-associated genes, genomic alterations occurring in genes not included in the panel could not be detected and therefore may have remained uncharacterized.

The requirement for available PD-L1 analysis may have introduced a degree of selection bias in the final cohort composition. In addition, no formal correction for multiple comparisons was applied, and therefore, some statistically significant findings should be interpreted cautiously. Although our findings are consistent with previously published studies and independent cohorts reported in the literature, the present study does not include a formal external validation cohort. Future prospective multicenter studies with larger cohorts and standardized integration of molecular and clinical data may help to further validate and expand these findings.

In conclusion, this study provides a real-world integrative analysis of lung cancer molecular profiles using next-generation sequencing, confirming previously described co-mutation patterns across histological subtypes. In particular, our findings corroborate the enrichment of *KRAS* mutations in adenocarcinoma and the relative molecular exclusivity of *EGFR*-mutated tumors, supporting their established role as major oncogenic drivers. Additionally, the lack of association between genomic alterations and PD-L1 expression underscores the complexity of immune biomarker regulation. Overall, these findings reinforce the clinical utility of NGS as a comprehensive diagnostic tool in precision oncology and contribute to a better understanding of the molecular heterogeneity of lung cancer.

Given the exploratory nature of the study, the relatively limited sample size, the multiple subgroup comparisons performed using Fisher’s exact tests, and the absence of formal correction for multiple comparisons, the reported statistical associations should be interpreted cautiously and considered as hypothesis-generating rather than definitive evidence of causality.

## Figures and Tables

**Table 1:** Number of cases of the different histological subtypes of lung cancer.

Histological Subtype	N^er^ of Cases
Adenocarcinoma	65
Squamous cell c.	25
Neuroendocrine c.	2
Adenosquamous c.	2
Anaplastic c.	1
NOS	1
**Total**	**96**

**Table 2:** Distribution of mutations in absolute numbers according to histological subtype.

**Histological Type**	**N^er^ of** **Cases**	**Absent**	***TP53***	***KRAS***	***EGFR***	***BRAF***	***FGFR1***	***ALK***	***MDM2***
Adenocarcinoma	65	13	19	29	7	3	1	3	2
Adenosquamous c.	2	1		1					
Anaplastic c.	1		1	1					
Squamous cell c.	25	14	8	2	3		1		
Neuroendocrine c.	2	1			1				
NOS	1	1							
**Total**	**96**	**30**	**28**	**33**	**11**	**3**	**2**	**3**	**2**
**Histological Type**	**N^er^ of** **Cases**		***MET***	***PTEN***	***ARID1A***	***PIK3CA***	***TSC1***	***ATM***	***AKT***
Adenocarcinoma	65		2	2	2	2		1	1
Adenosquamous c.	2								
Anaplastic c.	1								
Squamous cell c.	25			1			1		
Neuroendocrine c.	2								
NOS	1								
**Total**	**96**		**2**	**3**	**2**	**2**	**1**	**1**	**1**

**Table 3:** Association between the KRAS mutation and histological subtype (Fisher’s exact test).

Histological Type	No *KRAS*	*KRAS*	Total
Adenocarcinoma	36	29	65
Squamous cell c.	23	2	25
Neuroendocrine c.	2	0	2
Adenosquamous c.	1	1	2
Anaplastic c.	0	1	1
NOS	1	0	1
**Total**	**63**	**33**	**96**
**Fisher’s exact test**			***p* = 0.001**

**Table 4:** Association between the absence of mutations and histological subtype (Fisher’s exact test).

Histological Type	Mutation	Absence	Total
Adenocarcinoma	52	13	65
Adenosquamous c.	1	1	2
Anaplastic c.	1	0	1
Squamous cell c.	11	14	25
Neuroendocrine c.	1	1	2
NOS	0	1	1
**Total**	**66**	**30**	**96**
**Fisher’s exact test**			***p* = 0.002**

**Table 5:** Distribution of the number of mutations by histological subtype (Fisher’s exact test).

Histological Type	Absence	1 Mutation	≥2 Mutations	Total
Adenocarcinoma	13	34	18	65
Adenosquamous c.	1	1	0	2
Anaplastic c.	0	0	1	1
Squamous cell c.	14	8	3	25
Neuroendocrine c.	1	1	0	2
NOS	1	0	0	1
**Total**	**30**	**44**	**22**	**96**
**Fisher’s exact test**				***p* = 0.054**

**Table 6:** Co-mutations of 2 mutations.

Histological Type	*TP53* + *MET*	*TP53* + *KRAS*	*TP53* + *ALK*	*TP53* + *BRAF*	*TP53* + *EGFR*	*KRAS* + *ATM*	*KRAS* + *PIK3CA*	*MDM2* + *MET*
Adenocarcinoma	1	8	1	1	1	1	1	1
Anaplastic c.		1						
Squamous cell c.					1			

**Table 7:** Co-mutations of 3 mutations.

Histological Type	*TP53* + *KRAS* + *TSC1*	*TP53* + *KRAS* + *PIK3CA*	*TP53* + *PTEN* + *FGFR1*	*TP53* + *PTEN* + *ARID1A*
Adenocarcinoma		1		1
Anaplastic c.				
Squamous cell c.	1		1	

**Table 8:** Co-mutations of 4 mutations.

Histological Type	*TP53* + *PTEN* + *ARID1A* + *BRAF*
Adenocarcinoma	1

**Table 9:** Association between *KRAS* + *TP53* co-mutation and histological subtype (Fisher’s exact test).

Histological Type	No *KRAS* + *TP53*	*KRAS* + *TP53*	Total
Adenocarcinoma	56	9	65
Adenosquamous c.	2	0	2
Anaplastic c.	0	1	1
Squamous cell c.	24	1	25
Neuroendocrine c.	2	0	2
NOS	1	0	1
**Total**	**85**	**11**	**96**
**Fisher’s exact test**			***p* = 0.184**

**Table 10:** Distribution of co-mutations in tumors with an *EGFR* mutation (Fisher’s exact test).

*EGFR* Mutation	*EGFR*	Co-Mutation	Total
Absent	85	0	85
Present	9	2	11
**Total**	**94**	**2**	**96**
**Fisher’s exact test**			***p* = 0.012**

**Table 11:** Distribution of PD-L1 expression.

PD-L1 Expression	Number of Cases	% of Total
1–49%	42	43.8%
<1%	41	42.7%
≥50%	13	13.5%
**Total**	**96**	**100%**

**Table 12:** Association between PD-L1 expression and histological subtype.

Histological Type	PD-L1			Total
	1–49%	<1%	≥50%	
Adenocarcinoma	23	31	11	65
Adenosquamous c.	2	0	0	2
Anaplastic c.	1	0	0	1
Squamous cell c.	15	8	2	25
Neuroendocrine c.	0	2	0	2
NOS	1	0	0	1
**Total**	**42**	**41**	**13**	**96**
**Fisher’s exact test**				***p* = 0.185**

**Table 13:** Association between the *TP53* mutation and PD-L1 expression.

*TP53*	PD-L1			Total
	1–49%	<1%	≥50%	
Absent	30	30	8	68
Present	12	11	5	28
**Total**	**42**	**41**	**13**	**96**
**Fisher’s exact test**				***p* = 0.744**

**Table 14:** Association between the *KRAS* mutation and PD-L1 expression.

*KRAS*		PD-L1		Total
	1–49%	<1%	≥50%	
Absent	27	29	7	63
Present	15	12	6	33
**Total**	**42**	**41**	**13**	**96**
**Fisher’s exact test**				***p* = 0.508**

**Table 15:** Association between the *EGFR* mutation and PD-L1 expression.

*EGFR*		PD-L1		Total
	1–49%	<1%	≥50%	
Absent	38	34	13	85
Present	4	7	0	11
**Total**	**42**	**41**	**13**	**96**
**Fisher’s exact test**				***p* = 0.242**

**Table 16:** Multivariate binomial logistic regression analysis evaluating independent associations with PD-L1 expression.

Variable	OR	95% CI	*p*
*KRAS*	1.677	0.642–4.380	0.291
*TP53*	1.136	0.451–2.860	0.786
*EGFR*	0.438	0.110–1.740	0.240
Adenocarcinoma	0.426	0.162–1.120	0.083

## Data Availability

The original contributions presented in this study are included in the article. Further inquiries can be directed to the corresponding authors.

## Appendix A

Associations between various mutations and histological subtypes of lung cancer.

The following tables show the contingency tables for the remaining genes analyzed, with no significant differences according to Fisher’s exact test.

**Table A1:** Association between the TP53 mutation and histological subtype (Fisher’s exact test).

Histological Type	No *TP53*	*TP53*	Total
Adenocarcinoma	46	19	65
Adenosquamous	2	0	2
Anaplastic c.	0	1	1
Squamous cell c.	17	8	25
Neuroendocrine c.	2	0	2
NOS	1	0	1
**Total**	**68**	**28**	**96**
**Fisher’s exact test**			***p* = 0.678**

**Table A2:** Association between the EGFR mutation and histological subtype (Fisher’s exact test).

Histological Type	No *EGFR*	*EGFR*	Total
Adenocarcinoma	58	7	65
Adenosquamous	2	0	2
Anaplastic c.	1	0	1
Squamous cell c.	22	3	25
Neuroendocrine c.	1	1	2
NOS	1	0	1
**Total**	**85**	**11**	**96**
**Fisher’s exact test**			***p* = 0.559**

**Table A3:** Association between the BRAF mutation and histological subtype (Fisher’s exact test).

Histological Type	No *BRAF*	*BRAF*	Total
Adenocarcinoma	62	3	65
Adenosquamous	2	0	2
Anaplastic c.	1	0	1
Squamous cell c.	25	0	25
Neuroendocrine c.	2	0	2
NOS	1	0	1
**Total**	**93**	**3**	**96**
**Fisher’s exact test**			***p* = 0.636**

**Table A4:** Association between the FGFR1 mutation and histological subtype (Fisher’s exact test).

Histological Type	No *FGFR1*	*FGFR1*	Total
Adenocarcinoma	64	1	65
Adenosquamous	2	0	2
Anaplastic c.	1	0	1
Squamous cell c.	24	1	25
Neuroendocrine c.	2	0	2
NOS	1	0	1
**Total**	**94**	**2**	**96**
**Fisher’s exact test**			***p* = 0.544**

**Table A5:** Association between the ALK mutation and histological subtype (Fisher’s exact test).

Histological Type	No *ALK*	*ALK*	Total
Adenocarcinoma	61	4	65
Adenosquamous	2	0	2
Anaplastic c.	1	0	1
Squamous cell c.	25	0	25
Neuroendocrine c.	2	0	2
NOS	1	0	1
**Total**	**92**	**4**	**96**
**Fisher’s exact test**			***p* = 0.671**

**Table A6:** Association between the MDM2 mutation and histological subtype (Fisher’s exact test).

Histological Type	No *MDM2*	*MDM2*	Total
Adenocarcinoma	63	2	65
Adenosquamous	2	0	2
Anaplastic c.	1	0	1
Squamous cell c.	25	0	25
Neuroendocrine c.	2	0	2
NOS	1	0	1
**Total**	**94**	**2**	**96**
**Fisher’s exact test**			***p* = 1.000**

**Table A7:** Association between the MET mutation and histological subtype (Fisher’s exact test).

Histological Type	No *MET*	*MET*	Total
Adenocarcinoma	63	2	65
Adenosquamous	2	0	2
Anaplastic c.	1	0	1
Squamous cell c.	25	0	25
Neuroendocrine c.	2	0	2
NOS	1	0	1
**Total**	**94**	**2**	**96**
**Fisher’s exact test**			***p* = 1.000**

**Table A8:** Association between the ARID1A mutation and histological subtype (Fisher’s exact test).

Histological Type	No *ARID1A*	*ARID1A*	Total
Adenocarcinoma	63	2	65
Adenosquamous	2	0	2
Anaplastic c.	1	0	1
Squamous cell c.	25	0	25
Neuroendocrine c.	2	0	2
NOS	1	0	1
**Total**	**94**	**2**	**96**
**Fisher’s exact test**			***p* = 1.000**

**Table A9:** Association between the PTEN mutation and histological subtype (Fisher’s exact test).

Histological Type	No *PTEN*	*PTEN*	Total
Adenocarcinoma	63	2	65
Adenosquamous	2	0	2
Anaplastic c.	1	0	1
Squamous cell c.	24	1	25
Neuroendocrine c.	2	0	2
NOS	1	0	1
**Total**	**93**	**3**	**96**
**Fisher’s exact test**			***p* = 1.000**

**Table A10:** Association between the PIK3CA mutation and histological subtype (Fisher’s exact test).

Histological Type	No *PIK3CA*	*PIK3CA*	Total
Adenocarcinoma	63	2	65
Adenosquamous	2	0	2
Anaplastic c.	1	0	1
Squamous cell c.	25	0	25
Neuroendocrine c.	2	0	2
NOS	1	0	1
**Total**	**94**	**2**	**96**
**Fisher’s exact test**			***p* = 1.000**

**Table A11:** Association between the TSC1 mutation and histological subtype (Fisher’s exact test).

Histological Type	No *TSC1*	*TSC1*	Total
Adenocarcinoma	65	0	65
Adenosquamous	2	0	2
Anaplastic c.	1	0	1
Squamous cell c.	24	1	25
Neuroendocrine c.	2	0	2
NOS	1	0	1
**Total**	**95**	**1**	**96**
**Fisher’s exact test**			***p* = 0.323**

**Table A12:** Association between the ATM mutation and histological subtype (Fisher’s exact test).

Histological Type	No *ATM*	*ATM*	Total
Adenocarcinoma	64	1	65
Adenosquamous	2	0	2
Anaplastic c.	1	0	1
Squamous cell c.	25	0	25
Neuroendocrine c.	2	0	2
NOS	1	0	1
**Total**	**95**	**1**	**96**
**Fisher’s exact test**			***p* = 1.000**

**Table A13:** Association between the AKT mutation and histological subtype (Fisher’s exact test).

Histological Type	No *AKT*	*AKT*	Total
Adenocarcinoma	64	1	65
Adenosquamous	2	0	2
Anaplastic c.	1	0	1
Squamous cell c.	25	0	25
Neuroendocrine c.	2	0	2
NOS	1	0	1
**Total**	**95**	**1**	**96**
**Fisher’s exact test**			***p* = 1.000**
